# Predicting decay pathways in superheavy nuclei: theoretical insights into $\alpha$ and cluster radioactivity

**DOI:** 10.1038/s41598-025-33593-4

**Published:** 2026-02-05

**Authors:** M. Ismail, A. Adel, A. Y. Ellithi, Alaa Khaled

**Affiliations:** Physics Department, Faculty of Science, Cairo University, 12613 Giza, Egypt

**Keywords:** Chemistry, Physics

## Abstract

We employ the density-dependent cluster model to calculate $\alpha$-decay half-lives of recently synthesized superheavy nuclei (SHN) with $Z=104$–118. A microscopic $\alpha$–nucleus potential is derived via the double-folding method using a realistic nucleon–nucleon interaction. Within the Wentzel–Kramers–Brillouin approximation, supplemented by the Bohr–Sommerfeld quantization condition, we extract both the $\alpha$-particle assault frequency and barrier-penetration probability for spherical and deformed daughter configurations. Our predictions for five isotopes of the superheavy element $Z=123$ are benchmarked against several established models, demonstrating excellent agreement. We also explore the competition between $\alpha$-decay and spontaneous fission, and propose likely decay chains for the as-yet unobserved nuclei ${}^{302\text {--}307}123$. Finally, cluster-decay channels of ${}^{300,303,306,307}123$ are studied using the double-folding potential alongside the Universal curve (UNIV), the Universal Decay Law (UDL), the Unified Decay Formula (UDF), and Horoi’s approach. Notably, the UDL framework predicts positive branching ratios $\log _{10}b_c$ for heavy-cluster emission (e.g. $^{90}\textrm{Sr}$, $^{96}\textrm{Zr}$, $^{102}\textrm{Mo}$), indicating that such clusters may rival—or even dominate—$\alpha$-decay in these SHN.

## Introduction

The exploration of new superheavy elements (SHEs) and the quest for the anticipated “island of stability” have been central themes in modern nuclear physics^1–5^. In this transuranic region, strong quantum shell effects—most notably at the neutron magic number $N=184$ and a still–debated proton shell between $Z=114$ and $Z=126$—are believed to generate enhanced fission barriers, thereby extending lifetimes far beyond liquid-drop expectations. To date, the heaviest synthesized nuclide is $^{294}_{118}\textrm{Og}$ (half-life $0.89^{+1.07}_{-0.31}\,\textrm{ms}$) produced via $^{48}\textrm{Ca}+^{249}\textrm{Cf}$ fusion^6^, yet it remains several neutrons less of the predicted $N=184$ closure. Ongoing campaigns targeting $Z=119$ and $Z=120$ isotopes (e.g. $^{58}\textrm{Fe}+^{244}\textrm{Pu}$, $^{50}\textrm{Ti}+^{249}\textrm{Bk}$) face precipitously low cross sections ($<0.1\,$pb) and formidable target-fabrication challenges^7^. Under these circumstances, robust theoretical guidance is indispensable for optimizing beam–target combinations, designing decay-identification schemes, and charting the boundaries of superheavy element formation.

Superheavy nuclei typically undergo chains of successive $\alpha$ decays before terminating in spontaneous fission. Because each $\alpha$ emission carries precise information on decay energy and half-life, it serves as a sensitive probe of nuclear properties such as isospin asymmetry, shell closures, neutron-skin thickness, and deformation, and remains the primary signature for discovering new SHEs^8–17^. In neutron-rich superheavy isotopes, heavier-cluster emission—an analogous tunneling process—can also compete with $\alpha$ decay and fission, offering alternative decay routes as highlighted by Poenaru *et al.*^18–20^.

A fully microscopic description of $\alpha$ decay and cluster radioactivity remains a challenging quantum-mechanical problem, and over the past decades a variety of approaches have been proposed. Hartree–Fock–Bogoliubov calculations with the Gogny interaction have been used to study cluster emission in actinides^21^, while energy-density-functional methods have yielded microscopic half-lives for the $^{108}\textrm{Xe}\rightarrow {}^{104}\textrm{Te}\rightarrow {}^{100}\textrm{Sn}$ chain^22^ and time-dependent pairing models have addressed fine-structure effects in $\alpha$ decay^23^. The quartetting wave-function approach^11,24^ and generator-coordinate method^25^ have further elucidated cluster preformation, bridging fission-like and particle-emission pictures. Complementing these are semi-empirical frameworks—such as the generalized liquid-drop^26,27^, density-dependent cluster^28,29^, and Coulomb–proximity potential (CPPM) models^30^—and extensive systematics of $\alpha$- and cluster-decay half-lives using mass models and empirical formulas^31,32^. These theoretical foundations motivate our focused survey of decay modes in $Z=123$ isotopes.

Recent systematic studies have proposed to advance the understanding of cluster and $\alpha$-decay processes in superheavy nuclei. Manjunatha *et al.*^33^ studied the competition between $\alpha$, cluster, and fission decay modes in the $Z=126$ region within the proximity potential framework, demonstrating that $\alpha$ decay remains the dominant decay mode for the studied isotopes of superheavy nuclei. Sridhar *et al.*^34^ have studied the cluster radioactivity of actinides in the atomic number range $89< Z < 103$ in the framework of a WKB approximation. Nagaraja *et al.*^35,36^ applied modified generalized liquid-drop (MGLDM) and Coulomb-proximity potential (CPPM) models with various proximity functions to describe cluster and $\alpha$-decay half-lives for $105\le Z\le 125$, achieving close agreement with experiment and highlighting the sensitivity of decay predictions to the chosen interaction potential. Motivated by these developments, the present work focuses on the $\alpha$- and cluster-decay characteristics of the yet-unobserved nucleus with $Z = 123$ within the microscopic density-dependent cluster model (DDCM).

It is noteworthy that the superheavy element with proton number $Z = 123$ occupies a particularly significant position within the region of the predicted island of stability. Theoretical investigations based on macroscopic–microscopic models and self-consistent mean-field approaches predict that nuclei near $Z \approx 120$–126 and $N \approx 184$ may possess enhanced shell stability arising from strong spin–orbit coupling and relativistic shell effects. The investigation of nuclei with $Z = 123$ is therefore of particular importance, as it provides a crucial test for the persistence of nuclear binding and the predicted shell closures at extreme proton numbers. Furthermore, the study of their decay characteristics—especially $\alpha$ and cluster emissions—offers valuable insight into the underlying shell structure, deformation effects, and the limits of nuclear stability. Reliable theoretical predictions for $Z = 123$ thus play an essential role in guiding ongoing experimental efforts to synthesize elements beyond $Z = 120$ and to identify their possible production channels and decay chains, thereby contributing to a deeper understanding of the structure and stability of superheavy nuclei.

The present work extends decay-mode predictions to the as-yet unsynthesized element $Z=123$ (isotopes $300 \le A \le 307$). We compute $\alpha$-decay half-lives using the density-dependent cluster model, obtain barrier-penetration probabilities via the WKB approximation with Bohr–Sommerfeld quantization, and derive $\alpha$-preformation factors from the cluster-formation model^37,38^. These results are benchmarked against several empirical formulas and compared with spontaneous-fission lifetimes to determine the dominant decay channel for each isotope. We further assess the feasibility of heavier-cluster emission using complementary theoretical frameworks, and examine how nuclear deformation influences both $\alpha$ and cluster-decay half-lives. Our results identify isotopes with $\alpha$ chains, delineate the boundary where fission dominates, and highlight candidate cluster emitters in neutron-rich regimes. These predictions form a practical roadmap for forthcoming experimental campaigns, offering guidance on the most promising isotopes, expected decay energies, and lifetimes for detector planning.

The paper is structured as follows. In Sec.2 we outline the theoretical formalism used to compute $\alpha$- and cluster-decay half-lives. Sec.3 then presents and interprets our numerical results, and Sec. 4 summarizes the main findings and conclusions.

## Theoretical Framework

### Cluster decay in the density-dependent cluster model

We treat the parent nucleus as a two-body system of a preformed cluster and daughter, with the total potential

1 $$\begin{aligned} V_{\textrm{T}}(R,\theta ) =\lambda (\theta )\,V_{N}(R,\theta ) +V_{C}(R,\theta ) +\frac{\hbar ^2}{2\mu }\frac{(\ell +\tfrac{1}{2})^2}{R^2}\,, \end{aligned}$$

where $\lambda (\theta )$ renormalizes the double-folding nuclear potential, $R$ is the cluster–daughter separation, $\theta$ the emission angle, and $\mu$ their reduced mass. The Langer-corrected centrifugal term $\bigl (\ell +\tfrac{1}{2}\bigr )^2$ ensures proper behavior at $R\rightarrow 0$^39,40^. The minimum orbital angular momentum $\ell$ follows from

2 $$\begin{aligned} |J_i - J_f|\le \ell \le J_i + J_f,\qquad \frac{\pi _f}{\pi _i}=(-1)^{\ell }\,, \end{aligned}$$

Here, $J_i$ ($\pi _i$) and $J_f$ ($\pi _f$) denote the spin (parity) of the parent and daughter nuclei, respectively, with ground-state $J^\pi$taken from Ref^41^.. If either spin or parity is not known experimentally, we default to $\ell =0$.

Within the double-folding approach, both nuclear and Coulomb potentials are given by

3 $$\begin{aligned} V_{N,C}(R,\theta ) =\int d^3r_1\!\int d^3r_2\;\rho _c(r_1)\;v_{N,C}(s)\;\rho _d(r_2)\, \end{aligned}$$

Here, $\vec {s} = \vec {r}_2 - \vec {r}_1 + \vec {R}$ is the separation vector between a nucleon in the emitted cluster and one in the daughter nucleus, and $v_C(s)$ denotes the standard point-Coulomb interaction between protons. For the nuclear part $v_N(s)$, we adopt the M3Y–Paris three-range Yukawa potential with an added zero-range exchange term.

4 $$\begin{aligned} \begin{aligned} \upsilon _N(s) =&\left[ 11061.625\, \frac{e^{-4s}}{4\,s}-2537.5\,\frac{e^{-2.5s}}{2.5\,s}\right] \\&-590 \left[ 1-0.002\left( \frac{E_c}{A_c}\right) \right] \,\delta (\vec {s}), \end{aligned} \end{aligned}$$

where the cluster kinetic energy is

$$\begin{aligned} E_c=\frac{A_d\,Q_c}{A_c+A_d}\, \end{aligned}$$

and $Q_c$ follows from mass excesses^42^. The delta function term $\delta (\vec {s})$, in Eq. (4), denotes the zero–range pseudo–potential that represents the knock–on exchange part of the effective M3Y–Paris nucleon–nucleon interaction^43,43^. This delta function simulates the exchange of identical nucleons between the interacting cluster and daughter nuclei, which arises naturally from the antisymmetrization of their total wave function. This treatment effectively reproduces the knock–on exchange effects while preserving the analytical simplicity of a local potential and has been successfully employed in numerous double–folding calculations of $\alpha$ and cluster decay^29,44–46^.

The matter- and charge-density profiles of deformed nuclei are described with the standard two–parameter Fermi (2pF) form,

5 $$\begin{aligned} \rho (r,\theta ') \;=\; \frac{\rho _{0}}{1+\exp \!\bigl [(r-R(\theta '))/a\bigr ]}, \end{aligned}$$

where $\theta '$ is the angle between the position vector $\vec {r}$ and the nuclear symmetry axis. The normalisation constant $\rho _{0}$ is fixed so that the density integrates to the mass or charge of the nucleus. The orientation–dependent half–density radius is taken as

6 $$\begin{aligned} R(\theta ') = R_{0}\!\left[ 1 + \beta _{2}Y_{20}(\theta ') + \beta _{4}Y_{40}(\theta ')\right] , \end{aligned}$$

with $\beta _{2}$ and $\beta _{4}$the quadrupole and hexadecapole deformations adopted from Ref^47^.. We use

7 $$\begin{aligned} R_{0}=1.07\,A_{d}^{1/3}\;\text {fm}, \qquad a = 0.54\;\text {fm}, \end{aligned}$$

where $A_{d}$ is the daughter mass number; for spherical nuclei $R(\theta ')$ reduces to $R_{0}$.

The renormalisation factor $\lambda (\theta )$ in the total potential, Eq. (1), is *not* adjusted: it is fixed for each decay by enforcing the Bohr–Sommerfeld condition^48,49^,

8 $$\begin{aligned} \int _{R_{1}(\theta )}^{R_{2}(\theta )} k(r,\theta )\,dr \;=\; \frac{(2n+1)\pi }{2} \;=\; \frac{(G-\ell +1)\pi }{2}, \end{aligned}$$

with

9 $$\begin{aligned} k(r,\theta ) = \sqrt{\frac{2\mu \,|V_{T}(r,\theta )-Q_{c}|}{\hbar ^{2}}}. \end{aligned}$$

Here $R_{i}(\theta )$ ($i=1,2,3$) are the classical turning points defined by $V_{T}=Q_{c}$. The principal quantum number $G=2n+\ell$ is obtained from the Wildermuth–Tang condition^28,50,51^,

10 $$\begin{aligned} G =\sum _{i=1}^{A_{c}} \Bigl [g_{i}^{(A_{c}+A_{d})}-g_{i}^{(A_{c})}\Bigr ], \end{aligned}$$

where the oscillator quantum numbers $g_{i}$ ensure that the cluster nucleons occupy shells outside those filled by the core. In this work we set $g_{i}(50\!\le \!Z,N\!\le \!82)=4$, $g_{i}(82\!<\!Z,N\!\le \!126)=5$, $g_{i}(126\!<\!N\!\le \!184)=6$, and $g_{i}(N\!>\!184)=7$, corresponding to the $4\hbar \omega$–$7\hbar \omega$ shells.

It is noteworthy that the microscopic double-folding potential obtained from the folding of the nucleon densities of the emitted cluster and the daughter nucleus with the M3Y effective nucleon–nucleon interaction is generally too deep to reproduce experimental decay widths directly. This behavior arises because the simple folding formalism does not fully account for antisymmetrization and Pauli-blocking effects between nucleons of the cluster and those of the core. To correct for these missing exchange correlations, the nuclear part of the potential is multiplied by a renormalization factor, $\lambda$, which slightly reduces its depth to a physically realistic value. Importantly, $\lambda$ is not treated as an empirical fitting parameter but is determined self-consistently by imposing the Bohr–Sommerfeld quantization condition in Eq. (8), ensuring that the quasi-bound cluster–core system supports the correct number of radial nodes corresponding to the occupied oscillator quanta of the composite configuration. This quantization-based normalization, originally introduced in the density-dependent cluster model by Xu and Ren^44,46^ and further supported by Kelkar *et al.*^40^ and Seif *et al.*^52^, provides a robust microscopic justification for the renormalization procedure and guarantees physically consistent quasi-bound states and realistic decay widths without the need for empirical adjustment.

For a fixed emission angle $\theta$ the knocking frequency and penetrability in the WKB approximation are

11 $$\begin{aligned} \nu (\theta )&=\left[ \int _{R_{1}(\theta )}^{R_{2}(\theta )} \frac{2\mu }{\hbar \,k(r,\theta )}\,dr \right] ^{-1}, \end{aligned}$$

12 $$\begin{aligned} P(\theta )&=\exp \!\bigl [ -2\int _{R_{2}(\theta )}^{R_{3}(\theta )} k(r,\theta )\,dr \bigr ]. \end{aligned}$$

The partial width at angle $\theta$ is $\Gamma (\theta )=\hbar \,S_{c}\,\nu (\theta )\,P(\theta )$, and the total width is obtained by averaging over all orientations:

13 $$\begin{aligned} \Gamma =\frac{1}{2}\int _{0}^{\pi } \Gamma (\theta )\,\sin \theta \,d\theta . \end{aligned}$$

The cluster preformation factor is taken as^29,53^

14 $$\begin{aligned} \log _{10}S_{c} = a\,\sqrt{\mu \,Z_{c}Z_{d}} + b, \end{aligned}$$

with $a=-0.05663$ and $b=-0.02201$. Finally, the half–life is $T_{1/2}=\hbar \ln 2/\Gamma .$

### Density–dependent cluster model for $\alpha$ decay

The density–dependent cluster model^12,28,44–46,54^ successfully describes both cluster radioactivity and $\alpha$ decay. When it is applied to $\alpha$ emission, the cluster density $\rho _{c}$ in Eq. (3) (usually a two–parameter Fermi form) is replaced by the intrinsic density of the $\alpha$ particle,

15 $$\begin{aligned} \rho _{\alpha }(r_{1}) = 0.4229\, \exp \!\bigl (-0.7024\,r_{1}^{2}\bigr ), \end{aligned}$$

whose volume integral equals the mass number $A_{\alpha }=4$.

In the present calculations, the global quantum number *G* appearing in the Bohr–Sommerfeld quantization condition represents the total number of oscillator quanta associated with the relative motion between the emitted cluster and the daughter nucleus. It is determined by the Wildermuth-Tang condition^44–46,48,50,52^. This condition ensures that the cluster nucleons occupy orbits above those filled in the core, thereby respecting the Pauli exclusion principle. The global quantum number *G* that appears in Eq. (8) is chosen as^44–46,48^

$$\begin{aligned} G = {\left\{ \begin{array}{ll} 20, & N>126,\\ 18, & 82 < N \le 126,\\ 16, & N \le 82. \end{array}\right. } \end{aligned}$$

These values correspond to major shell closures and have been shown to reproduce experimental systematics of $\alpha$ decay across a wide range of medium, heavy, and superheavy nuclei.

The *Q*-values for $\alpha$-decay are taken from the WS4+ mass model with radial-basis-function corrections (WS4+ RBF)^55^. The WS4 model represents the latest refinement of the Weizsäcker–Skyrme macroscopic–microscopic mass formula and incorporates a surface diffuseness correction that significantly improves the description of symmetry energy and shell effects, particularly for nuclei approaching the drip lines. This enhancement reduces the global root-mean-square (rms) deviation of nuclear masses to about 298 keV and yields a precision better than 300 keV for $Q_{\alpha }$ values of superheavy nuclei. The RBF approach further refines the predictive accuracy by globally minimizing the residual discrepancies between theoretical and experimental masses. A comprehensive benchmarking study by Wang *et al.*^31^ systematically evaluated *Q*-values of nuclei ($Z \ge 100$) using 20 global mass models. Their results demonstrated that the WS4 model provided the smallest deviation from experimental $Q_{\alpha }$ values, outperforming FRDM, KTUY, DZ, and HFB models. Therefore, the WS4 + RBF model was selected in this work as it offers a superior combination of global consistency and local accuracy for heavy and superheavy nuclei.

The $\alpha$ preformation factor $S_{\alpha }$ is evaluated with the Cluster Formation Model (CFM)^37,38^:

16 $$\begin{aligned} S_{\alpha } = \frac{\langle \phi _{f}|H_{f}|\phi _{f}\rangle }{\langle \phi _{f}|H_{f}|\phi _{f}\rangle + \langle \phi _{r}|H_{r}|\phi _{r}\rangle }, \end{aligned}$$

where $\phi _{f}$ and $H_{f}$ refer to cluster formation, and $\phi _{r}$ and $H_{r}$ to the relative motion of the $\alpha$ and daughter. Solving $H_{f}\phi _{f}=E_{f}\phi _{f}$ and $H_{r}\phi _{r}=E_{r}\phi _{r}$, with $E=E_{f}+E_{r}$, gives $S_{\alpha }=E_{f}/E=E_{\alpha }/E .$. The explicit expressions for $E_{\alpha }$ and *E* depend on the neutron (*N*) and proton (*Z*)^38^:

**even** *N*, **even** *Z*:

17 $$\begin{aligned} E_{\alpha }(N_{e},Z_{e})&= 3\,B(N_{e},Z_{e}) + B(N_{e}\!-\!2, Z_{e}\!-\!2) \nonumber \\&\quad - 2\,B(N_{e}, Z_{e}\!-\!1) - 2\,B(N_{e}\!-\!1, Z_{e}), \nonumber \\ E(N_{e},Z_{e})&= B(N_{e},Z_{e}) - B(N_{e}\!-\!2,Z_{e}\!-\!2); \end{aligned}$$

**odd** *N*, **even** *Z*:

18 $$\begin{aligned} E_{\alpha }(N_{o},Z_{e})&= 3\,B(N_{o}\!-\!1, Z_{e}) + B(N_{o}\!-\!3, Z_{e}\!-\!2) \nonumber \\&\quad - 2\,B(N_{o}\!-\!1, Z_{e}\!-\!1) - 2\,B(N_{o}\!-\!2, Z_{e}), \nonumber \\ E(N_{o},Z_{e})&= B(N_{o},Z_{e}) - B(N_{o}\!-\!3,Z_{e}\!-\!2); \end{aligned}$$

**even** *N*, **odd** *Z*:

19 $$\begin{aligned} E_{\alpha }(N_{e},Z_{o})&= 3\,B(N_{e}, Z_{o}\!-\!1) + B(N_{e}\!-\!2, Z_{o}\!-\!3) \nonumber \\&\quad - 2\,B(N_{e}, Z_{o}\!-\!2) - 2\,B(N_{e}\!-\!1,Z_{o}\!-\!1), \nonumber \\ E(N_{e},Z_{o})&= B(N_{e},Z_{o}) - B(N_{e}\!-\!2,Z_{o}\!-\!3); \end{aligned}$$

**odd** *N*, **odd** *Z*:

20 $$\begin{aligned} E_{\alpha }(N_{o},Z_{o})&= 3\,B(N_{o}\!-\!1,Z_{o}\!-\!1) + B(N_{o}\!-\!3,Z_{o}\!-\!3) \nonumber \\&\quad - 2\,B(N_{o}\!-\!1,Z_{o}\!-\!2) - 2\,B(N_{o}\!-\!2,Z_{o}\!-\!1), \nonumber \\ E(N_{o},Z_{o})&= B(N_{o},Z_{o}) - B(N_{o}\!-\!3,Z_{o}\!-\!3). \end{aligned}$$

Here *B*(*N*, *Z*) is the binding energy of the nucleus (*N*, *Z*).

The angle–dependent knocking frequency and penetrability are

21 $$\begin{aligned} \nu (\theta )&= \biggl [ \int _{R_{1}(\theta )}^{R_{2}(\theta )} \frac{2\mu }{\hbar \,k(r,\theta )}\,dr \biggr ]^{-1}, \nonumber \\ P(\theta )&= \exp \!\bigl [ -2\int _{R_{2}(\theta )}^{R_{3}(\theta )} k(r,\theta )\,dr \bigr ], \end{aligned}$$

and the total decay width is

$$\begin{aligned} \Gamma = \frac{1}{2} \int _{0}^{\pi }\! \hbar \,S_{\alpha }\,\nu (\theta )\,P(\theta )\; \sin \theta \,d\theta . \end{aligned}$$

Finally, the half–life follows from $T_{1/2}=\hbar \ln 2/\Gamma$.

### Empirical Formulas

#### Viola–Seaborg–Sobiczewski (VSS)

The Viola–Seaborg relation, with parameters updated by Sobiczewski *et al.*^56^, reads

22 $$\begin{aligned} \log _{10}T_{1/2}^{\text {VSS}} =(aZ+b)Q_{\alpha }^{-1/2}+cZ+d+h_{\log }, \end{aligned}$$

where $T_{1/2}$ is in seconds, $Q_{\alpha }$ in MeV. In our work, we have adopted the constants provided by Sobiczewski *et al.*^56^: $a = 1.66175,\quad b = -8.5166,\quad c = -0.20228,\quad d = -33.9069,$ The hindrance term is

$$\begin{aligned} h_{\log } = {\left\{ \begin{array}{ll} 0 & Z,N\text { even},\\ 0.772 & Z\text { odd},\,N\text { even},\\ 1.066 & Z\text { even},\,N\text { odd},\\ 1.114 & Z,N\text { odd}. \end{array}\right. } \end{aligned}$$

#### Universal Curve (UNIV)

Poenaru *et al.*^20^ proposed the universal relation

23 $$\begin{aligned} \log _{10}T = -\log _{10}P_{S} - \log _{10}S + \bigl [\log _{10}(\ln 2)-\log _{10}\nu \bigr ], \end{aligned}$$

with $\nu$ the assault frequency, *S* the cluster preformation factor, and $P_{S}$ the Coulomb penetrability between the turning points $R_{t}=R_{d}+R_{e}$ and $R_{b}=1.43998\,Z_{d}Z_{e}/Q$:

$$\begin{aligned} -\log _{10}P_{S}=0.22873\sqrt{\mu _{A}Z_{d}Z_{e}R_{b}} \bigl [\arccos \sqrt{r}-\sqrt{r(1-r)}\bigr ], \end{aligned}$$

where $r=R_{t}/R_{b}$, $R_{t}=1.2249\,(A_{d}^{1/3}+A_{e}^{1/3})$ fm. The preformation factor is $\log _{10}S=-0.598\,(A_{e}-1)$, and for even–even parents $c_{ee}=-\log _{10}\nu +\log _{10}(\ln 2)=-22.16917$.

#### Universal Decay Law (UDL)

Qi *et al.*^57^ derived a linear relation in *R*-matrix theory,

24 $$\begin{aligned} \log _{10}T_{1/2} =a\,Z_{c}Z_{d}\sqrt{\frac{A}{Q_{c}}} +b\,\sqrt{A\,Z_{c}Z_{d}(A_{d}^{1/3}+A_{c}^{1/3})} +c, \end{aligned}$$

where $A=A_{d}A_{c}/(A_{d}+A_{c})$. The fitted constants are $a=0.3949,\;b=-0.3693,\;c=-23.7615$.

#### Royer

Within the generalised liquid-drop picture, Royer^58^ obtained

25 $$\begin{aligned} \log _{10}T_{1/2}(\text {s}) = a + b\,A^{1/6}\sqrt{Z} + \frac{cZ}{\sqrt{Q_{\alpha }}}, \end{aligned}$$

with parameter sets

$$\begin{aligned} (a,b,c)= {\left\{ \begin{array}{ll} (-25.31,-1.1629,1.5864) & Z\text { even},N\text { even},\\ (-26.65,-1.0859,1.5848) & Z\text { even},N\text { odd},\\ (-25.68,-1.1423,1.5920) & Z\text { odd}, N\text { even},\\ (-29.48,-1.1130,1.6971) & Z\text { odd},N\text { odd}.\\ \end{array}\right. } \end{aligned}$$

#### Modified Brown prescription (mB1)

Budaca *et al.*^59^ generalised Brown’s half-life expression by allowing the power of $Z_{d}$ to be fitted, yielding

26 $$\begin{aligned} \log _{10}T_{1/2}^{\text {mB1}} = a\,(Z-2)^{\,b}\,Q_{\alpha }^{-1/2} + c + h_{\text {mB1}}, \end{aligned}$$

with $(a,b,c)=(13.0705,\,0.5182,\,-47.8867)$. The hindrance factor is

$$\begin{aligned} h_{\text {mB1}}= {\left\{ \begin{array}{ll} 0 & Z,N\text { even},\\ 0.6001 & Z\text { odd},\,N\text { even},\\ 0.4666 & Z\text { even},\,N\text { odd},\\ 0.8200 & Z,N\text { odd}. \end{array}\right. } \end{aligned}$$

#### Horoi scaling rule

Horoi *et al.*^60^ proposed a scaling relation in which the half-life varies linearly with $(Z_{c}Z_{d})^{0.6}/Q_{c}$ and with $\sqrt{\mu }$:

27 $$\begin{aligned} \log _{10}T_{1/2}^{\text {H}} = (a_{1}\sqrt{\mu }+b_{1})\Bigl [(Z_{\alpha }Z_{d})^{0.6}Q^{-1/2}-7\Bigr ] +(a_{2}\sqrt{\mu }+b_{2}), \end{aligned}$$

where $$a_{1}=6.8$$, $$b_{1}=-7.5$$, $$a_{2}=6.9$$, and $$b_{2}=-22.4$$.

#### Unified Decay Formula (UDF)

Derived from a simplified WKB treatment, the UDF of Ni *et al.*^61^ reads

28 $$\begin{aligned} \log _{10}T_{1/2} = c\,Z_{c}Z_{d}\sqrt{\frac{\mu }{Q_{c}}} + d\,\sqrt{\mu Z_{c}Z_{d}} + h, \end{aligned}$$

with $\mu =A_{c}A_{d}/(A_{c}+A_{d})$. In the case of cluster emission the coefficients are $c=0.38617, d=-1.08676$ and $h=-20.11223$.

### Spontaneous-fission (SF) systematics

Yuan *et al.*^62^ recently proposed an updated SF half-life formula for $Z\ge 108$:

29 $$\begin{aligned} \begin{aligned} \log _{10}\!\left( T_{1/2}^{\text {SF}}\right) =&\; 28.60 + c_{1}\frac{Z-90-v}{A} + c_{2}\frac{(Z-90-v)^{2}}{A} \\&+ c_{3}\frac{|Z-90-v|^{1/2}(N-Z-52)^{2}}{A} \\&+ c_{4}\frac{(Z-90-v)(N-152)^{2}}{A} \\&+ c_{5}\frac{(Z-90-v)^{2}(N-162)}{A} \\&+ c_{6}\frac{|Z-90-v|^{5/2}|N-162|^{3/2}}{A}, \end{aligned} \end{aligned}$$

The fitted constants are $c_{1}=-646.834955$, $c_{2}=10.058292$, $c_{3}=-11.472012$, $c_{4}=0.698861$, $c_{5}=-0.209631$, and $c_{6}=-0.016230$. In this expression we take $v=0$ for even–even nuclei and $v=2$ for odd-$A$ or odd–odd parents.

## Results and discussions

We investigated the newly synthesised super-heavy nuclei $104\le Z\le 118$ within the density–dependent cluster model (DDCM). All input $Q_{\alpha }$ values were taken from the most recent mass evaluation AME2020^63^, while the experimental half-lives were adopted from the evaluated data set NUBASE2020^41^. The $\alpha$–daughter interaction was constructed microscopically with a double–folding procedure that employs the M3Y–Paris nucleon–nucleon force. Two shape options were considered for the daughter nucleus: spherical (Sph.) and axially deformed (Def.) through the tabulated quadrupole and hexadecapole parameters $\beta _{2}$ and $\beta _{4}$. For each case the action integral was evaluated with the WKB method, and the Bohr–Sommerfeld quantisation condition was imposed to fix the renormalisation factor in the nuclear potential.

**Fig. 1:**
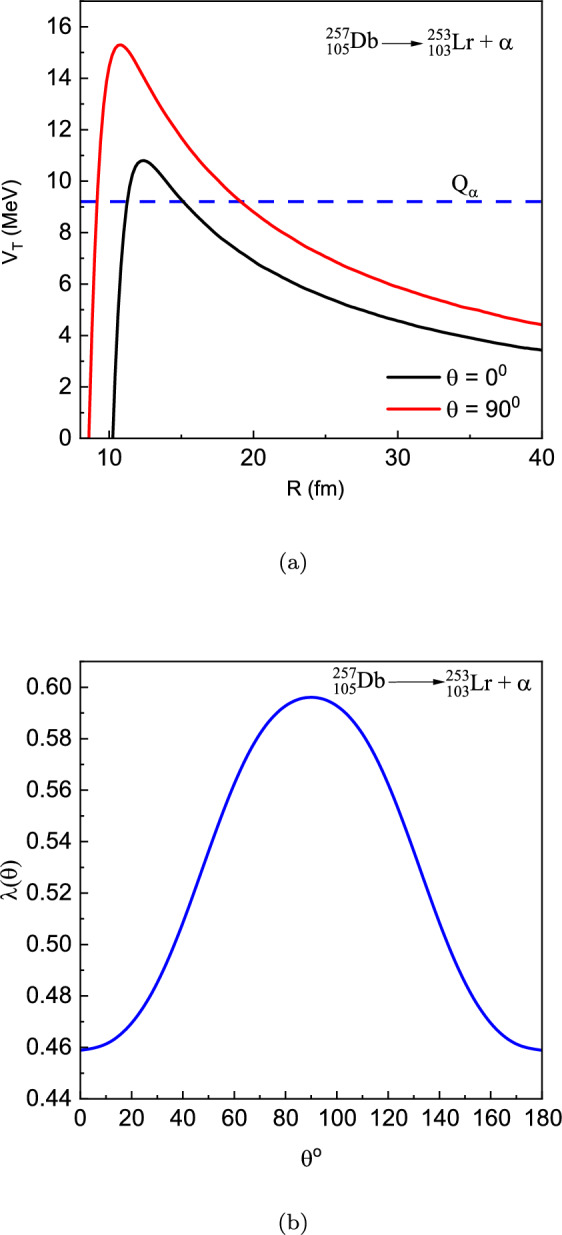
(**a**) The total interaction potential $V_T(R,\theta )$ for the $\alpha$-decay of $^{257}\textrm{Db} \rightarrow \, ^{253}\textrm{Lr} + \alpha$ calculated at two orientation angles of the deformed daughter nucleus, $\theta = 0^{\circ }$ and $\theta = 90^{\circ }$. (**b**) The corresponding variation of the renormalization factor $\lambda (\theta )$ obtained from the Bohr–Sommerfeld quantization condition.

Figure 1(a) illustrates the total interaction potential $V_T(R,\theta )$ for the $\alpha$-decay of $^{257}\textrm{Db} \rightarrow \, ^{253}\textrm{Lr} + \alpha$ at two orientation angles of the deformed daughter nucleus, $\theta = 0^{\circ }$ and $\theta = 90^{\circ }$. The potential exhibits the typical barrier structure dominated by the Coulomb repulsion and the attractive nuclear component, with the barrier height being noticeably larger at $\theta = 90^{\circ }$ than at $\theta = 0^{\circ }$. This angular dependence arises from the deformation of the daughter nucleus, which modifies the overlap between the cluster and core densities in the double-folding potential. Figure 1(b) presents the corresponding variation of the renormalization factor $\lambda (\theta )$ with the orientation angle. The parameter $\lambda$, which represents the renormalization of the microscopic nuclear folding potential, is determined from the Bohr–Sommerfeld (BS) quantization condition. This condition ensures that the quasi-bound cluster–core system satisfies the correct semiclassical quantization rule, thereby fixing the depth of the nuclear potential. The BS condition guarantees that the potential supports the correct number of radial nodes corresponding to the occupied oscillator quanta, providing a microscopic normalization rather than an empirical adjustment. As shown in Fig. 1(b), $\lambda (\theta )$ increases smoothly from $\lambda = 0.4589$ at $\theta = 0^{\circ }$ to $\lambda = 0.5961$ at $\theta = 90^{\circ }$, reflecting the enhanced nuclear attraction for orientations with larger surface overlap. The relative increase of $\lambda$ between these two orientations is approximately $29.9\%$. This variation indicates that nuclear deformation alters the height and curvature of the potential barrier, which modifies the quantum tunneling probability and, in turn, influences the calculated decay half-life.

**Fig. 2:**
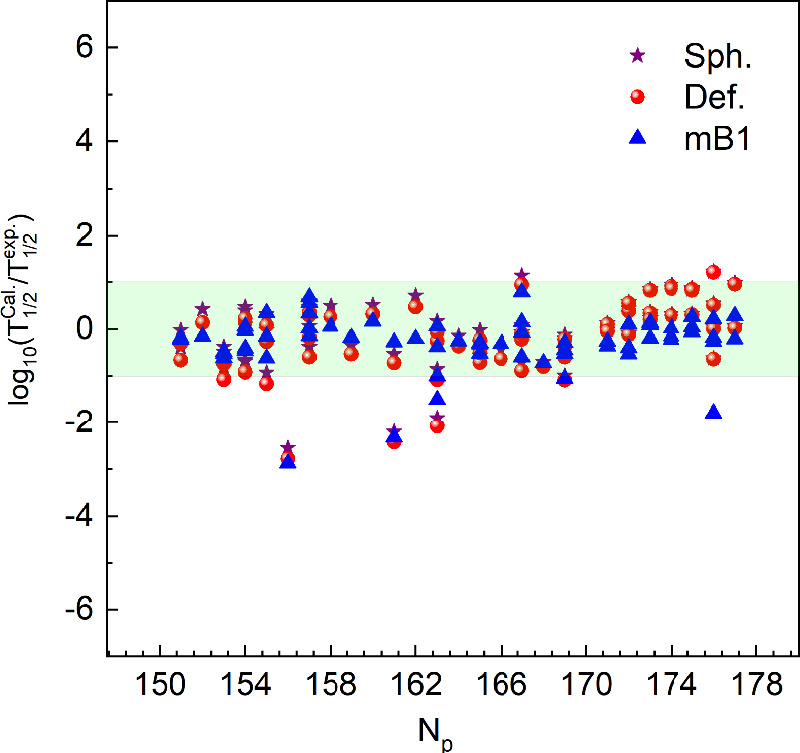
Deviation between the calculated $\alpha$-decay half-life and the corresponding experimental value listed in Table 2. $T_{1 / 2}^{\text{ Sph. } }$, $T_{1 / 2}^{\text{ Def. } }$ denote the theoretical $\alpha$-decay half-lives calculated with the double-folding potential and $T_{1 / 2}^{\text{ mB1 }}$.

The analysis of the angular dependence of the potential and the normalization of the nuclear interaction provides the basis for extending the calculations to the complete set of superheavy nuclei under investigation. The resulting half-lives for both spherical and deformed configurations are summarised in Table 1, together with predictions from several widely used empirical formulas, including VSS^56^,, Royer^58^,, UNIV^20^,, UDL^57^,, and the modified Brown formula (mB1)^59^,.

**Table 1:** Comparison of the predicted^41^$\alpha$ -decay half-lives with various theoretical models; The half-lives are calculated using the double-folding model based on M3Y-Paris*NN* (Sph.) assuming spherical shape of the daughter nuclei. The calculation of half-lives with including deformation of the daughter nuclei (Def.) are also added for comparison, as well as by Viola and Seaborg(VSS)^56^, Royer^58^ and Universal curve of Poenaru et al.(UNIV)^20^. Also The Universal Decay Law (UDL)^57^, Modified Brown formula(mB1)^59^, and An improved formula for spontaneous fission^62^.The Q-values are obtained from the most recent mass evaluation AME2020^63^.

Parent Nuclei	$Q_\alpha ^{\text {Expt.}}$ (MeV)	$T_{1/2}^{\alpha }\text {(s)}$								SF
		Exp.	Sph.	Def.	VSS	Royer	UNIV	UDL	mB1	
$_{ 118 }^{ 294 } \text { Og }$	11.870	7.00E-04	2.43E-03	2.29E-03	4.64E-04	1.53E-04	2.81E-04	1.66E-04	4.63E-04	3.05E+08
$_{ 117 }^{ 294 } \text { Ts }$	11.180	7.00E-02	7.95E-02	7.64E-02	1.38E-01	2.87E-03	7.25E-02	3.55E-03	4.31E-02	1.03E+04
$_{ 117 }^{ 293 } \text { Ts }$	11.320	2.50E-02	2.76E-02	2.62E-02	2.84E-02	1.39E-03	6.89E-03	1.67E-03	1.35E-02	1.22E+05
$_{ 116 }^{ 293 } \text { Lv }$	10.680	7.00E-02	6.77E-01	6.54E-01	1.18E+00	2.46E-02	2.90E-01	3.31E-02	1.34E-01	3.66E+00
$_{ 116 }^{ 292 } \text { Lv }$	10.791	1.60E-02	2.65E-01	2.53E-01	5.20E-02	1.34E-02	2.81E-02	1.76E-02	2.63E-02	6.65E+01
$_{ 116 }^{ 291 } \text { Lv }$	10.890	2.60E-02	1.93E-01	1.84E-01	3.36E-01	7.88E-03	8.94E-02	1.02E-02	4.74E-02	8.00E+02
$_{ 116 }^{ 290 } \text { Lv }$	11.000	9.00E-03	7.77E-02	7.39E-02	1.52E-02	4.40E-03	8.94E-03	5.57E-03	9.52E-03	6.39E+03
$_{ 115 }^{ 290 } \text { Mc }$	10.410	8.40E-01	1.75E+00	1.64E+00	3.43E+00	6.53E-02	2.00E+00	9.09E-02	7.34E-01	8.77E-01
$_{ 115 }^{ 289 } \text { Mc }$	10.490	4.10E-01	8.38E-01	7.87E-01	9.48E-01	4.19E-02	2.23E-01	5.75E-02	2.93E-01	1.07E+01
$_{ 115 }^{ 288 } \text { Mc }$	10.650	1.77E-01	4.08E-01	3.81E-01	7.83E-01	1.69E-02	4.49E-01	2.25E-02	2.16E-01	8.71E+01
$_{ 115 }^{ 287 } \text { Mc }$	10.760	6.00E-02	1.64E-01	1.53E-01	1.84E-01	9.26E-03	4.70E-02	1.21E-02	7.52E-02	4.68E+02
$_{ 114 }^{ 290 } \text { Fl }$	9.860	8.00E+01	1.90E+01	1.75E+01	4.47E+00	9.70E-01	2.19E+00	1.46E+00	1.30E+00	4.37E-05
$_{ 114 }^{ 289 } \text { Fl }$	9.950	2.10E+00	1.44E+01	1.33E+01	2.85E+01	5.61E-01	6.51E+00	8.30E-01	2.31E+00	1.24E-03
$_{ 114 }^{ 288 } \text { Fl }$	10.076	6.53E-01	4.97E+00	4.58E+00	1.07E+00	2.60E-01	5.70E-01	3.77E-01	3.96E-01	2.32E-02
$_{ 114 }^{ 287 } \text { Fl }$	10.170	5.10E-01	3.55E+00	3.26E+00	6.79E+00	1.49E-01	1.68E+00	2.13E-01	6.99E-01	2.88E-01
$_{ 114 }^{ 286 } \text { Fl }$	10.360	2.20E-01	8.25E-01	6.54E-01	1.75E-01	4.83E-02	1.02E-01	6.67E-02	8.76E-02	2.37E+00
$_{ 114 }^{ 285 } \text { Fl }$	10.560	2.10E-01	2.79E-01	2.24E-01	5.95E-01	1.52E-02	1.60E-01	2.02E-02	9.20E-02	1.30E+01
$_{ 113 }^{ 286 } \text { Nh }$	9.790	1.20E+01	2.03E+01	1.66E+01	4.38E+01	8.12E-01	2.77E+01	1.21E+00	7.64E+00	1.19E-03
$_{ 113 }^{ 285 } \text { Nh }$	10.010	4.60E+00	3.77E+00	3.03E+00	4.64E+00	2.04E-01	1.07E+00	2.94E-01	1.36E+00	1.51E-02
$_{ 113 }^{ 284 } \text { Nh }$	10.280	9.70E-01	9.32E-01	7.50E-01	1.82E+00	3.97E-02	9.97E-01	5.44E-02	5.33E-01	1.26E-01
$_{ 113 }^{ 282 } \text { Nh }$	10.780	1.40E-01	3.82E-02	2.68E-02	8.86E-02	2.31E-03	4.27E-02	2.87E-03	4.25E-02	2.58E+00
$_{ 112 }^{ 281 } \text { Cn }$	10.430	1.80E-01	1.35E-01	9.14E-02	3.13E-01	8.54E-03	7.92E-02	1.12E-02	6.58E-02	7.20E-02
$_{ 112 }^{ 277 } \text { Cn }$	11.620	7.90E-04	2.17E-04	1.35E-04	4.02E-04	1.69E-05	1.20E-04	1.68E-05	2.43E-04	1.20E+00
$_{ 111 }^{ 280 } \text { Rg }$	10.149	4.30E+00	4.38E-01	2.89E-01	9.56E-01	2.23E-02	4.56E-01	3.01E-02	3.85E-01	2.40E-03
$_{ 111 }^{ 279 } \text { Rg }$	10.530	1.70E-01	3.41E-02	2.13E-02	4.27E-02	2.45E-03	1.03E-02	3.07E-03	3.27E-02	1.38E-02
$_{ 111 }^{ 278 } \text { Rg }$	10.850	8.00E-03	6.88E-03	4.28E-03	1.47E-02	4.28E-04	5.66E-03	4.99E-04	1.13E-02	5.24E-02
$_{ 111 }^{ 274 } \text { Rg }$	11.480	2.00E-02	2.54E-04	1.56E-04	4.79E-04	1.90E-05	1.71E-04	1.91E-05	6.28E-04	1.79E-01
$_{ 111 }^{ 272 } \text { Rg }$	11.197	4.20E-03	1.24E-03	7.69E-04	2.15E-03	8.54E-05	9.29E-04	9.29E-05	2.23E-03	2.80E-02
$_{ 110 }^{ 273 } \text { Ds }$	11.370	2.40E-04	2.14E-04	1.29E-04	3.91E-04	1.71E-05	1.10E-04	1.72E-05	2.79E-04	9.02E-02
$_{ 110 }^{ 271 } \text { Ds }$	10.870	5.76E-01	3.53E-03	2.05E-03	5.86E-03	2.44E-04	1.79E-03	2.79E-04	2.78E-03	3.32E-02
$_{ 110 }^{ 270 } \text { Ds }$	11.117	2.05E-04	6.73E-04	3.92E-04	1.29E-04	6.89E-05	1.05E-04	7.45E-05	2.99E-04	1.09E-02
$_{ 110 }^{ 269 } \text { Ds }$	11.510	2.30E-04	1.05E-04	6.19E-05	1.89E-04	9.96E-06	6.28E-05	9.76E-06	1.51E-04	2.36E-03
$_{ 110 }^{ 267 } \text { Ds }$	11.780	1.00E-05	2.93E-05	1.74E-05	4.84E-05	2.95E-06	1.74E-05	2.71E-06	4.74E-05	3.22E-05
$_{ 109 }^{ 278 } \text { Mt }$	9.580	6.00E+00	3.85E+00	2.53E+00	8.47E+00	1.82E-01	3.98E+00	2.61E-01	3.11E+00	4.14E-06
$_{ 109 }^{ 276 } \text { Mt }$	10.100	7.00E-01	1.38E-01	8.79E-02	2.97E-01	7.58E-03	1.19E-01	9.90E-03	1.79E-01	4.97E-04
$_{ 109 }^{ 275 } \text { Mt }$	10.480	3.10E-02	1.14E-02	6.98E-03	1.37E-02	8.64E-04	3.24E-03	1.04E-03	1.53E-02	2.94E-03
$_{ 109 }^{ 266 } \text { Mt }$	10.996	2.00E-03	8.41E-04	4.85E-04	1.63E-03	7.41E-05	6.86E-04	8.08E-05	2.11E-03	2.41E-04
$_{ 108 }^{ 275 } \text { Hs }$	9.450	2.80E-01	3.79E+00	2.32E+00	8.39E+00	2.16E-01	1.78E+00	3.10E-01	1.71E+00	5.74E-05
$_{ 108 }^{ 273 } \text { Hs }$	9.650	1.06E+00	1.01E+00	6.00E-01	2.21E+00	6.34E-02	5.07E-01	8.83E-02	5.45E-01	3.13E-03
$_{ 108 }^{ 270 } \text { Hs }$	9.070	9.00E+00	4.59E+01	2.49E+01	1.02E+01	3.53E+00	7.17E+00	5.38E+00	5.68E+00	5.78E-02
$_{ 108 }^{ 267 } \text { Hs }$	10.038	6.88E-02	1.03E-01	5.81E-02	1.86E-01	7.22E-03	5.44E-02	9.43E-03	6.55E-02	2.63E-02
$_{ 108 }^{ 266 } \text { Hs }$	10.346	3.95E-03	1.23E-02	6.93E-03	2.48E-03	1.24E-03	2.03E-03	1.52E-03	4.53E-03	8.90E-03
$_{ 108 }^{ 265 } \text { Hs }$	10.470	1.96E-03	7.48E-03	4.24E-03	1.40E-02	6.39E-04	4.42E-03	7.65E-04	7.12E-03	1.99E-03
$_{ 108 }^{ 264 } \text { Hs }$	10.591	1.00E+00	2.81E-03	1.59E-03	5.98E-04	3.40E-04	5.33E-04	3.97E-04	1.34E-03	2.96E-04
$_{ 108 }^{ 263 } \text { Hs }$	10.730	9.00E-04	1.84E-03	1.03E-03	3.17E-03	1.66E-04	1.09E-03	1.88E-04	2.00E-03	2.91E-05
$_{ 107 }^{ 274 } \text { Bh }$	8.940	5.70E+01	6.95E+01	4.31E+01	1.52E+02	3.31E+00	8.15E+01	5.03E+00	4.86E+01	7.19E-06
$_{ 107 }^{ 272 } \text { Bh }$	9.300	1.13E+01	5.65E+00	3.35E+00	1.19E+01	2.94E-01	5.76E+00	4.24E-01	5.42E+00	9.21E-04
$_{ 107 }^{ 271 } \text { Bh }$	9.420	2.90E+00	2.08E+00	1.21E+00	2.39E+00	1.37E-01	5.79E-01	1.95E-01	1.62E+00	5.63E-03
$_{ 107 }^{ 270 } \text { Bh }$	9.060	2.28E+02	3.23E+01	1.82E+01	6.40E+01	1.65E+00	3.80E+01	2.47E+00	2.30E+01	2.28E-02
$_{ 107 }^{ 262 } \text { Bh }$	10.319	8.40E-02	9.99E-03	5.50E-03	1.83E-02	7.99E-04	8.43E-03	9.67E-04	2.05E-02	6.16E-04
$_{ 107 }^{ 261 } \text { Bh }$	10.500	1.28E-02	2.65E-03	1.45E-03	2.92E-03	3.02E-04	1.04E-03	3.52E-04	5.01E-03	6.15E-05
$_{ 107 }^{ 260 } \text { Bh }$	10.400	4.10E-02	6.31E-03	3.43E-03	1.14E-02	5.47E-04	5.53E-03	6.53E-04	1.36E-02	4.07E-06
$_{ 106 }^{ 269 } \text { Sg }$	8.580	3.00E+02	4.43E+02	2.52E+02	8.81E+02	2.44E+01	1.97E+02	3.80E+01	1.26E+02	1.67E-02
$_{ 106 }^{ 263 } \text { Sg }$	9.400	1.08E+00	1.26E+00	7.02E-01	2.47E+00	9.54E-02	6.97E-01	1.34E-01	7.76E-01	1.71E-01
$_{ 106 }^{ 261 } \text { Sg }$	9.714	1.87E-01	1.77E-01	9.64E-02	3.17E-01	1.40E-02	9.69E-02	1.86E-02	1.32E-01	1.38E-02
$_{ 106 }^{ 260 } \text { Sg }$	9.901	1.71E-02	4.17E-02	2.24E-02	8.41E-03	4.64E-03	7.66E-03	5.98E-03	1.63E-02	2.12E-03
$_{ 106 }^{ 259 } \text { Sg }$	9.765	4.02E-01	1.32E-01	6.99E-02	2.29E-01	1.10E-02	7.61E-02	1.46E-02	9.94E-02	2.15E-04
$_{ 105 }^{ 259 } \text { Db }$	9.620	5.10E-01	1.12E-01	6.06E-02	1.37E-01	1.23E-02	4.59E-02	1.64E-02	1.76E-01	7.53E-02
$_{ 105 }^{ 258 } \text { Db }$	9.437	3.39E+00	5.20E-01	2.76E-01	9.86E-01	4.09E-02	5.64E-01	5.61E-02	8.22E-01	1.17E-02
$_{ 105 }^{ 257 } \text { Db }$	9.206	2.45E+00	1.92E+00	9.95E-01	2.12E+00	1.94E-01	7.92E-01	2.76E-01	1.91E+00	3.74E+00
$_{ 105 }^{ 256 } \text { Db }$	9.340	2.43E+00	1.01E+00	5.26E-01	1.88E+00	8.30E-02	1.23E+00	1.16E-01	1.44E+00	8.29E-05
$_{ 104 }^{ 261 } \text { Rf }$	8.650	1.17E+01	4.36E+01	2.41E+01	9.72E+01	3.52E+00	2.48E+01	5.30E+00	2.54E+01	1.68E+01
$_{ 104 }^{ 258 } \text { Rf }$	9.196	2.55E-01	7.55E-01	4.04E-01	1.73E-01	8.79E-02	1.49E-01	1.23E-01	2.93E-01	2.71E+00
$_{ 104 }^{ 257 } \text { Rf }$	9.083	5.60E+00	2.36E+00	1.25E+00	4.36E+00	1.95E-01	1.30E+00	2.78E-01	1.69E+00	6.48E-01
$_{ 104 }^{ 256 } \text { Rf }$	8.926	2.13E+00	5.57E+00	2.90E+00	1.12E+00	5.97E-01	1.07E+00	8.69E-01	1.50E+00	1.03E-01
$_{ 104 }^{ 255 } \text { Rf }$	9.055	3.09E+00	2.95E+00	1.53E+00	5.29E+00	2.55E-01	1.72E+00	3.65E-01	2.00E+00	1.08E-02

To quantify the overall agreement we use the root-mean-square deviation of the decimal logarithms,

30 $$\begin{aligned} \sigma =\sqrt{\frac{1}{n-1}\sum _{i=1}^{n} \bigl (\log _{10}T_{1/2}^{\textrm{calc}} -\log _{10}T_{1/2}^{\textrm{expt}}\bigr )^{2}}, \end{aligned}$$

**Fig. 3:**
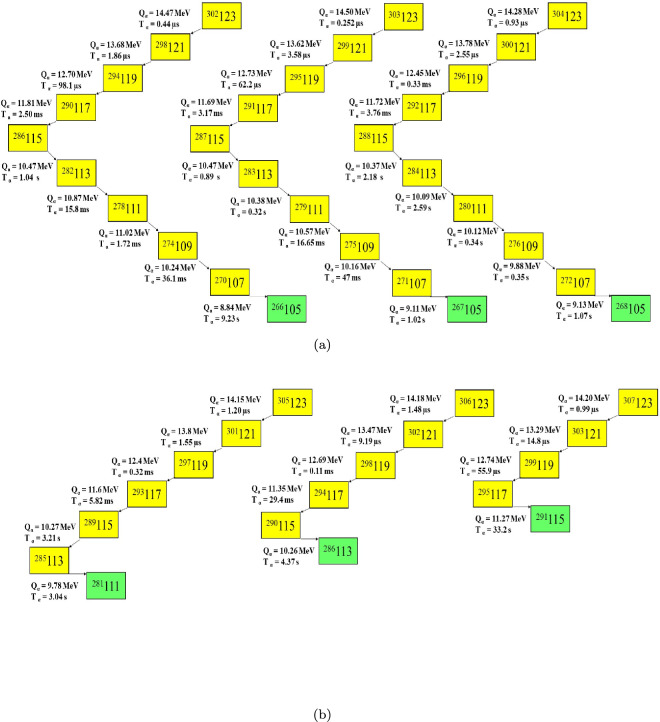
Predicted decay chains originating from $^{302-304}{123}$ isotopes. Yellow squares correspond to $\alpha$-decay, the green squares to spontaneous fission (SF) decay. The $\alpha$-decay energies calculated by $\text {WS4+}$ and $\alpha$-decay half lives with double-folding model (DFM) for deformed daughter.

Here, $n=66$ is the number of superheavy nuclei in the experimental dataset, and $\sigma$ denotes the root-mean-square deviation of the base-10 logarithms of the half-lives. The mB1 formula achieves the smallest $\sigma \approx 0.664$, indicating the closest overall agreement with experiment. In comparison, VSS yields $\sigma \approx 0.739$, Royer $\sigma \approx 0.799$, the spherical DFM $\sigma \approx 0.730$, and the deformed DFM $\sigma \approx 0.855$. The UDL and UNIV models perform worst, with $\sigma \approx 1.333$ and $\sigma \approx 1.424$, respectively, reflecting much poorer consistency. Figure 2 displays the deviations between the DFM and mB1 calculations of the $\alpha$-decay half-lives and the corresponding experimental values, underscoring the minimal scatter of the mB1 results. Figure 2 illustrates the quantity $\log _{10}\!\bigl (\frac{T_{1/2}^{\textrm{calc}}}{T_{1/2}^{\textrm{expt}}}\bigr )$ for the $\alpha$-decay half-lives of newly synthesized superheavy nuclei, plotted as a function of the parent neutron number $N_p$. Calculations using the double-folding model (DFM)—incorporating both spherical and deformed daughter configurations—closely reproduce the most recent experimental results and agree with the mB1 formula. Indeed, nearly all DFM points in Fig. 2 lie within the horizontal band $-1 \le \log_{10}\!\bigl (\tfrac{T_{1/2}^{\textrm{calc}}}{T_{1/2}^\textrm{expt}}\bigr) \le 1$. The predicted $\alpha$-decay chains of $^{302-307}123$ isotopes are presented in Table 2 and visually depicted in Fig. 3. In superheavy nuclei (SHN), $\alpha$-decay and spontaneous fission (SF) are the dominant decay modes. To determine the preferred decay modes of the decay chains of elements $^{302\text {--}307}123$, we have calculated both their $\alpha$-decay and SF half-lives. SF half-lives were obtained using the improved semi-empirical formula of Yuan *et al.*^62^, while $\alpha$-decay half-lives were derived from a double-folding model—incorporating spherical and deformed daughter shapes—augmented by five empirical relations (VSS, Royer, UNIV, UDL, and mB1). The resulting half-lives for $^{302\text {--}307}123$ and their decay chains are listed in Table 2. Predicted decay chains for the $^{302-307}123$ isotopes are illustrated in Fig. 3, where yellow squares mark $\alpha$-decay events and green squares denote spontaneous fission. The $\alpha$-decay energies were obtained with the WS4+ model^55^. Notably, the WS4$^+$ mass model is widely regarded as one of the most dependable for evaluating $Q_{\alpha }$ values in superheavy nuclei, achieving an accuracy better than 300 keV^55^. The corresponding half-lives for the deformed daughter nuclei were calculated using the double-folding model (DFM). Figure 3(a) displays the decay chains of $^{302-304}123$, whereas Figure 3(b) illustrates those of the heavier isotopes $^{305-307}123$. By highlighting the SF endpoints in green, Fig. 3 makes clear exactly where—and after how many $\alpha$ decays—spontaneous fission takes over for each $Z=123$ isotope.

**Fig. 4:**
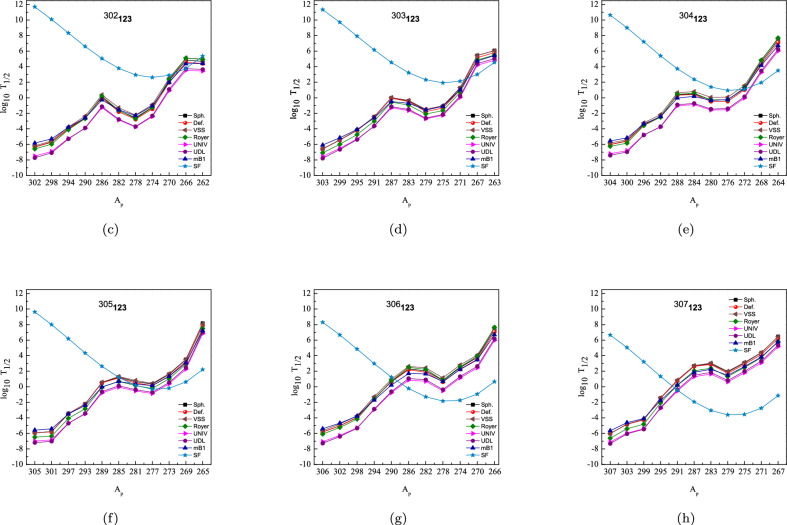
Comparison of the logarithmic $\alpha$-decay half-lives $\log _{10} T_{1/2}$ versus parent mass number $A_p$ for $^{302\text {--}307}123$ isotopes and their decay products, including spontaneous fission and other analytical models.

Figure 4 presents our calculated $\log _{10}T_{1/2}$ values for both $\alpha$-decay—using seven different models (spherical and deformed DFM, VSS, Royer, UNIV, UDL, and mB1)—and spontaneous fission (SF) across the six isotopes $^{302\text {--}307}123$. In each panel, the family of curves shows the variation of $\alpha$-decay half-lives with the parent mass number $A_p$, while the blue-star line denotes the SF half-life. The nucleus at which the SF line crosses below all of the $\alpha$-decay curves marks the end of that decay chain. For the lightest parent, $^{302}123$ (panel a), the elements $^{302}123$, $^{298}121$, $^{298}121$, $^{294}119$, $^{290}117$, $^{286}115$, $^{282}113$, $^{278}111$, $^{274}109$, and $^{270}107$ have an $\alpha$-decay half-life shorter than its SF half-life, and only after nine successive $\alpha$ emissions does the SF branch dominate at $^{266}105$. Thus, $^{302}123$ is predicted to survive fission and produce a nine-$\alpha$ decay chain. The very similar behavior seen in $^{303}123$ (panel b) and $^{304}123$ (panel c) likewise requires nine $\alpha$ steps to reach the SF terminus at $^{267}105$ and $^{268}105$, respectively. As we move to heavier parents, the chain length shrinks: six $\alpha$ emissions precede fission in $^{305}123$ (panel d), and five $\alpha$ chains in $^{306}123$ (panel e) and four $\alpha$ chains in $^{307}123$ (panel f). Two features stand out. First, each successive $\alpha$ emission shows a gradual drop in $Q_\alpha$ and a corresponding rise in $\log _{10}T_\alpha$, reflecting the lowering Coulomb barrier in lighter daughters. Second, all seven $\alpha$-decay models remain tightly grouped, demonstrating the robustness of both the DFM (spherical/deformed) and the empirical formulas across this mass region.

Beyond $\alpha$ decay, cluster radioactivity also represents a significant decay channel for heavy and superheavy nuclei. Cluster radioactivity, in which a parent nucleus emits a fragment heavier than an $\alpha$ particle yet lighter than typical fission fragments, provides a sensitive probe of shell effects and nuclear structure in the superheavy region. To date, only a limited set of cluster emissions—such as $^{14}\textrm{C}$, $^{20}\textrm{O}$, $^{22,24\text {--}26}\textrm{Ne}$, $^{28,30}\textrm{Mg}$, and $^{32,34}\textrm{Si}$—have been experimentally observed^64^. In Ref^65^., Poenaru *et al.* broadened the definition of cluster radioactivity to include emitted fragments with $Z_c>28$ from parent nuclei with $Z>110$.

They further predicted that, for certain synthesized superheavy isotopes, these heavier clusters would exhibit progressively shorter half-lives and higher branching ratios compared to $\alpha$ decay. Poenaru *et al.*^20,66^, using the superasymmetric fission model have shown that for superheavy nuclei with $Z \ge 121$, the cluster-emission branching ratio may exceed that of $\alpha$ decay, suggesting that cluster decay could dominate under suitable conditions. This remarkable prediction motivates a thorough theoretical survey of cluster-decay half-lives and branching ratios for isotopes near $Z=123$.

In the present study, we extend these analyses to the isotopes $^{300}123$, $^{303}123$, $^{306}123$, and $^{307}123$, calculating emission probabilities for candidate clusters such as $^{14}\textrm{C}$, $^{16}\textrm{O}$, $^{18}\textrm{O}$, $^{20}\textrm{O}$, $^{20}\textrm{Ne}$, $^{22}\textrm{Ne}$, $^{24}\textrm{Ne}$, $^{26}\textrm{Ne}$, $^{28}\textrm{Mg}$, $^{30}\textrm{Mg}$, $^{32}\textrm{Si}$, $^{34}\textrm{Si}$, $^{68}\textrm{Ni}$, $^{76}\textrm{Zn}$, $^{80}\textrm{Ge}$, $^{84}\textrm{Se}$, $^{86}\textrm{Kr}$, $^{90}\textrm{Sr}$, $^{92}\textrm{Sr}$, $^{94}\textrm{Sr}$, $^{96}\textrm{Zr}$, $^{102}\textrm{Mo}$. Our approach combines the density-dependent cluster model—utilizing an M3Y-based double-folding potential—with the deformation characteristics of the daughter nuclei, and employs $Q$-values derived from the WS4+ mass model with radial-basis-function corrections (WS4+ RBF)^63^. By comparing the resulting cluster-decay half-lives with competing $\alpha$-decay channel, we assess both the theoretical feasibility and the likely experimental observability of cluster radioactivity in these systems.

Table 3 summarizes the numerical cluster-decay half-lives calculated for the isotopes $^{300,303,306,307}123$.

**Table 2:** Calculated logarithmic $\alpha$-decay half-lives compared across several theoretical approaches. Results from the double-folding model (DFM) using the M3Y-Paris interaction for spherical daughter nuclei (Sph.) are shown alongside DFM predictions that include daughter-nucleus deformation (Def.). For comparison, half-lives from the Viola–Seaborg (VSS) scheme^56^, Royer’s formula^58^, the Universal curve of Poenaru *et al.* (UNIV)^20^, the Universal Decay Law (UDL)^57^, the Modified Brown formula (mB1)^59^, and an improved spontaneous-fission (SF) expression^62^ are also included. The *Q*-values are taken from the WS4+ mass model with radial-basis-function corrections (WS4+ RBF)^63^.

Parent Nuclei	$Q_\alpha ^{\text {WS4+}}$	$\log _{10}T_{1/2}^{\alpha }$							SF
	(MeV)	Sph.	Def.	VSS	Royer	UNIV	UDL	mB1	
$^{302} 123$	14.474	−6.327	−6.355	−6.187	−6.585	−7.489	−7.726	−5.829	11.702
$^{298} 121$	13.862	−5.695	−5.730	−5.551	−5.967	−6.909	−7.086	−5.291	10.085
$^{294} 119$	12.699	−3.979	−4.008	−3.762	−4.116	−5.243	−5.293	−3.801	8.335
$^{290} 117$	11.81	−2.576	−2.601	−2.363	−2.675	−3.914	−3.884	−2.601	6.608
$^{286} 115$	10.469	0.043	0.016	0.375	0.202	−1.274	−1.133	−0.267	5.049
$^{282} 113$	10.872	−1.649	−1.801	−1.284	−1.617	−2.859	−2.773	−1.565	3.791
$^{278} 111$	11.015	−2.561	−2.765	−2.235	−2.677	−3.753	−3.703	−2.286	2.949
$^{274} 109$	10.235	−1.219	−1.442	−0.886	−1.273	−2.435	−2.330	−1.054	2.623
$^{270} 107$	8.839	2.213	1.965	2.505	2.329	0.905	1.092	1.964	2.891
$^{266} 105$	7.872	4.849	4.579	5.121	5.109	3.536	3.745	4.373	3.757
$^{303} 123$	14.505	−6.577	−6.599	−6.187	−6.584	−7.098	−7.555	−6.093	11.335
$^{299} 121$	13.624	−5.412	−5.447	−5.551	−5.443	−5.984	−6.508	−5.147	9.708
$^{295} 119$	12.731	−4.177	−4.206	−3.762	−4.171	−4.735	−5.322	−4.076	7.929
$^{291} 117$	11.69	−2.444	−2.499	−2.363	−2.428	−3.009	−3.667	−2.594	6.158
$^{287} 115$	10.471	−0.023	−0.053	0.375	0.028	−0.563	−1.296	−0.491	4.544
$^{283} 113$	10.376	−0.397	−0.496	−1.284	−0.342	−0.946	−1.636	−0.710	3.222
$^{279} 111$	10.572	−1.575	−1.779	−2.235	−1.478	−2.095	−2.714	−1.577	2.312
$^{275} 109$	10.16	−1.111	−1.328	−0.886	−1.030	−1.652	−2.259	−1.105	1.920
$^{271} 107$	9.112	1.245	1.008	2.505	1.303	0.693	0.043	1.004	2.132
$^{267} 105$	7.697	5.445	5.178	5.121	5.448	4.869	4.186	4.735	3.007
$^{304} 123$	14.279	−6.015	−6.030	−5.837	−6.247	−7.199	−7.410	−5.548	10.643
$^{300} 121$	13.783	−5.557	−5.593	−5.403	−5.845	−6.803	−6.973	−5.171	9.011
$^{296} 119$	12.449	−3.451	−3.483	−3.232	−3.585	−4.775	−4.797	−3.369	7.209
$^{292} 117$	11.724	−2.401	−2.424	−2.165	−2.499	−3.758	−3.720	−2.439	5.400
$^{288} 115$	10.368	0.368	0.338	0.649	0.460	−1.042	−0.893	−0.039	3.738
$^{284} 113$	10.085	0.509	0.414	0.797	0.574	−0.880	−0.726	0.177	2.359
$^{280} 111$	10.123	−0.288	−0.468	0.051	−0.265	−1.583	−1.451	−0.354	1.390
$^{276} 109$	9.88	−0.258	−0.455	0.074	−0.279	−1.537	−1.403	−0.235	0.939
$^{272} 107$	9.134	1.256	1.027	1.578	1.299	−0.040	0.130	1.166	1.101
$^{268} 105$	7.937	4.602	4.341	4.878	4.812	3.260	3.468	4.162	1.949
$^{305} 123$	14.147	−5.912	−5.920	−5.837	−6.487	−6.992	−7.187	−5.575	9.630
$^{301} 121$	13.799	−5.777	−5.808	−5.403	−6.352	−6.846	−7.020	−5.415	7.998
$^{297} 119$	12.398	−3.455	−3.491	−3.232	−4.063	−4.687	−4.704	−3.499	6.179
$^{293} 117$	11.595	−2.213	−2.235	−2.165	−2.822	−3.487	−3.436	−2.411	4.340
$^{289} 115$	10.267	0.534	0.506	0.649	−0.040	−0.788	−0.632	−0.029	2.635
$^{285} 113$	9.782	1.234	1.138	0.797	0.688	−0.051	0.124	0.683	1.208
$^{281} 111$	9.734	0.663	0.483	0.051	0.159	−0.535	−0.373	0.350	0.187
$^{306} 123$	14.182	−5.823	−5.830	−5.660	−6.093	−7.066	−7.268	−5.406	8.300
$^{302} 121$	13.469	−5.019	−5.036	−4.802	−5.239	−6.277	−6.406	−4.686	6.675
$^{298} 119$	12.688	−3.942	−3.973	−3.739	−4.162	−5.284	−5.338	−3.782	4.845
$^{294} 117$	11.35	−1.515	−1.531	−1.277	−1.586	−2.943	−2.867	−1.709	2.981
$^{290} 115$	10.256	0.666	0.640	0.958	0.754	−0.775	−0.618	0.217	1.242
$^{286} 113$	9.464	2.289	2.200	2.620	2.488	0.867	1.062	1.702	−0.227
$^{307} 123$	14.201	−6.004	−6.005	−6.036	−6.622	−7.113	−7.319	−5.654	6.658
$^{303} 121$	13.287	−4.819	−4.831	−4.786	−5.398	−5.959	−6.066	−4.616	5.044
$^{299} 119$	12.739	−4.227	−4.253	−4.188	−4.824	−5.400	−5.461	−4.089	3.210
$^{295} 117$	11.27	−1.452	−1.479	−1.424	−2.075	−2.772	−2.689	−1.769	1.328
$^{291} 115$	10.166	0.785	0.761	0.868	0.206	−0.546	−0.383	0.206	−0.439

**Fig. 5:**
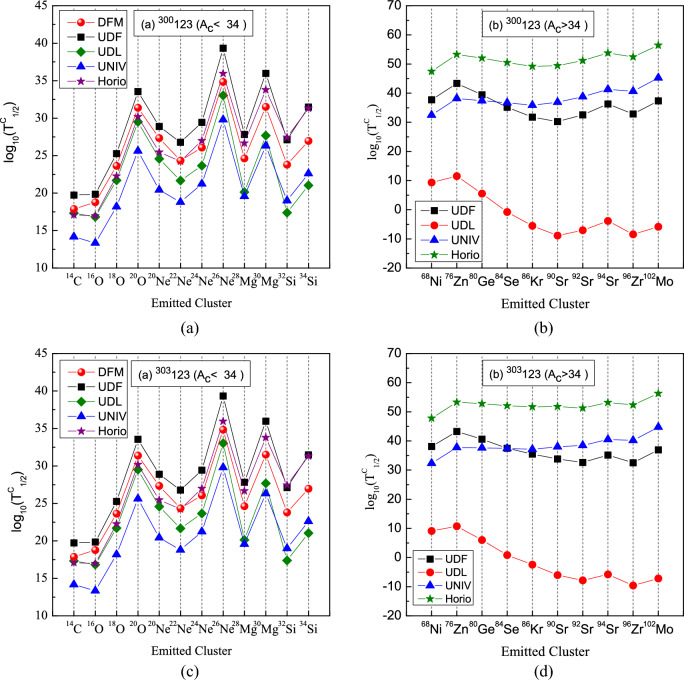
Comparison of logarithmic cluster-decay half-lives predicted by the double-folding model (DFM), the UDF, UDL, UNIV, and Horoi’s scaling law. Panels (**a**) and (**c**) correspond to cluster emissions with $A_c<34$ for the isotopes $^{300}123$ and $^{303}123$, respectively, while panels (**b**) and (**d**) depict emissions with $A_c>34$.

**Fig. 6:**
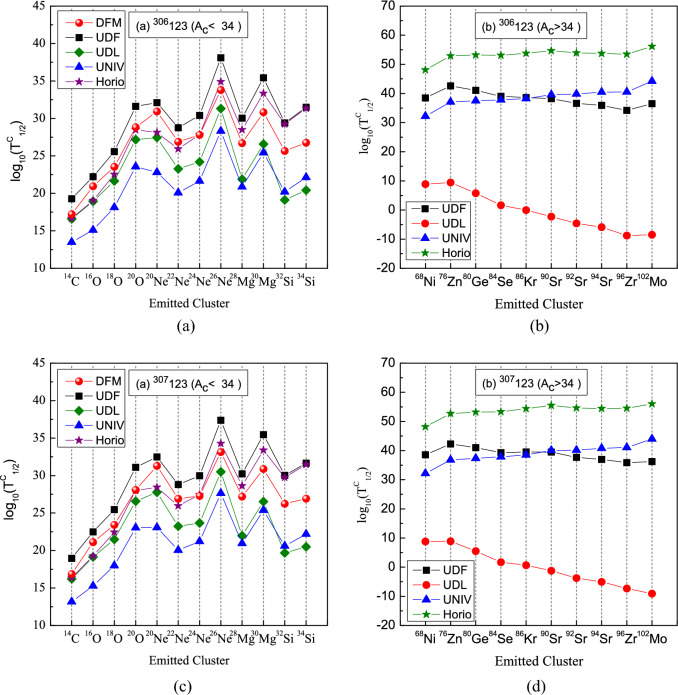
The same as Fig. 5 but for $^{306}123$ and $^{307}123$ isotopes.

Theoretical predictions from the double-folding model based on M3Y-Paris NN force considering deformation of daughter nuclei, as well as by Universal curve of Poenaru et al.(UNIV)^20^, the Universal Decay Law (UDL)^57^, the Unified decay formula(UDF)^61^, and Horoi formula^60^ are plotted in Figs. 5 and 6. Noticeable deviations among these approaches arise from the sparse experimental confirmation of cluster emissions, which introduces significant uncertainties in the parameters of the empirical formulas^64^. Despite these discrepancies, every method except UDL formula yields cluster half-lives that are orders of magnitude longer than the corresponding $\alpha$-decay half-lives, confirming that $\alpha$ decay remains the dominant mode for$^{300,303,306,307}123$.

Furthermore, as evident from Table 3 and panels (a) of Figs. 5 and 6, all models follow a consistent trend in the variation of half-lives for emitted clusters with $A_c<34$. Emissions with $A_c<34$ generally exhibit half-lives below $10^{30}\,\textrm{s}$, suggesting they lie within reach of experimental detection^67^. In particular, most such clusters from $^{300\text {--}302}123$ show half-lives under this threshold across all methods (with the exception of $^{20}$O, $^{26}$Ne and $^{30}$Mg).

A convenient way to quantify the competition between $\alpha$ decay and cluster radioactivity (CR) is via the branching ratio

31 $$\begin{aligned} \log _{10} b_{c} \;=\; \log _{10} T_{\alpha } \;-\; \log _{10} T_{c}\,. \end{aligned}$$

A positive value of $\log b_{c}$ indicates that cluster radioactivity outcompetes $\alpha$ decay, whereas a large negative $\log b_{c}$ implies that $\alpha$ decay—with its much shorter half-life—dominates. Table 3 summarizes the $\log _{10}b_{c}$ values computed using the double-folding model (DFM), accounting for daughter-nucleus deformations of multipolarities up to the hexacontratetrapole.

Table 3 reveals that the DFM, UDF, UNIV, and Horoi models all give $\log _{10}b_c\ll 0$, indicating that $\alpha$-decay half-lives are far shorter than those for cluster emission. By contrast, within the UDL framework, $\log _{10}b_c$ becomes positive for the heavy clusters $^{90}\textrm{Sr}$ and $^{96}\textrm{Zr}$ in $^{300}123$, for $^{92}\textrm{Sr}$ and $^{96}\textrm{Zr}$ in $^{303}123$, and for $^{96}\textrm{Zr}$ and $^{102}\textrm{Mo}$ in $^{306}123$ and $^{307}123$. This implies that these clusters can compete with—and may even dominate over—$\alpha$-decay in the studied superheavy nuclei under the UDL model. Within the UDL prescription, all cluster-decay half-lives listed in Table 3 lie below $10^{30}\,\textrm{s}$—with the sole exception of $^{26}\textrm{Ne}$—indicating that both light clusters ($^{14}\textrm{C},\,^{16}\textrm{O},\,^{18}\textrm{O},\,^{20}\textrm{O},\,^{20}\textrm{Ne},\,^{22}\textrm{Ne},\,^{24}\textrm{Ne},\,^{28}\textrm{Mg},\,^{30}\textrm{Mg},\,^{32}\textrm{Si}$) and heavier fragments ($^{68}\textrm{Ni},\,^{76}\textrm{Zn},\,^{80}\textrm{Ge},\,^{84}\textrm{Se},\dots$) could be within experimental reach. Remarkably, for $^{300}123$ the cluster-decay half-lives of $^{90}\textrm{Sr}$ and $^{96}\textrm{Zr}$ are shorter than their competing $\alpha$-decay half-lives, establishing cluster emission as the dominant mode in that case. Moreover, the shortest half-life predicted across all models corresponds to decay into the doubly-magic daughter $^{208}\textrm{Pb}$, underscoring the pivotal role of shell closures in enhancing cluster-decay rates. While the DFM, UDF, UNIV, and Horoi scaling laws generally favor lighter-cluster emission, the UDL framework instead predicts that heavier fragments will predominate.

**Table 3:** Comparison of the predicted logarithmic Cluster radioactivity half-lives with various theoretical models; The half-lives are calculated using the double-folding model based on M3Y-Paris , as well as by Universal curve of Poenaru et al.(UNIV)^20^ , The Universal Decay Law (UDL)^57^ , The Unified decay foemula(UDF)^61^ , and Horoi formula^60^ . The Q-values and the deformation parameters are extracted from the recent WS4+ mass model^63^ . Entries shown as dashed lines indicate that the double-folding model was unable to compute the cluster-decay half-lives for those cases, since their$Q_c$ values exceed the Coulomb barrier

Parent Nuclei	Cluster Nuclei	$\beta _{2}$	$\beta _{4}$	$\beta _{6}$	$Q_{c}^{\text {WS4+}}$	$\log _{10}T_{1/2}^{c}$					$\log _{10}b_{c}$
					(MeV)	DFM	UDF	UDL	UNIV	Horoi	
$_{ 177 }^{ 300 }{ 123 }$	$^{ 4 } \text { He }$	0.0695	-0.0304	0.0006	14.738	-6.792	-5.338	-8.154	-7.882	-6.947	-
	$^{ 14 } \text { C }$	0.0721	0.0043	-0.0041	46.621	17.880	19.742	17.334	14.174	17.100	-24.672
	$^{ 16 } \text { O }$	0.0618	0.0098	-0.0045	67.541	18.777	19.844	16.829	13.344	16.973	-25.569
	$^{ 18 } \text { O }$	-0.0573	0.0057	0.0003	65.221	23.632	25.254	21.694	18.180	22.260	-30.424
	$^{ 20 } \text { O }$	-0.0239	0.004	-0.0001	61.288	31.386	33.541	29.500	25.628	30.196	-38.178
	$^{ 20 } \text { Ne }$	0.1421	-0.0348	-0.0025	83.083	27.329	28.892	24.571	20.419	25.453	-34.121
	$^{ 22 } \text { Ne }$	0.1706	-0.0538	0.0011	86.514	24.342	26.770	21.659	18.803	24.252	-31.134
	$^{ 24 } \text { Ne }$	0.1839	-0.0566	0	85.920	26.066	29.439	23.649	21.239	27.001	-32.858
	$^{ 26 } \text { Ne }$	0.1849	-0.0502	-0.0014	80.196	34.825	39.321	33.021	29.787	35.943	-41.617
	$^{ 28 } \text { Mg }$	0.1981	-0.0503	-0.0043	109.410	24.633	27.820	20.128	19.573	26.666	-31.425
	$^{ 30 } \text { Mg }$	0.1923	-0.0418	-0.0041	103.935	31.507	35.963	27.674	26.308	33.779	-38.299
	$^{ 32 } \text { Si }$	0.2099	-0.0435	-0.0062	132.019	23.799	27.124	17.394	19.003	27.449	-30.591
	$^{ 34 } \text { Si }$	0.2201	-0.039	-0.0107	129.182	26.965	31.471	21.026	22.604	31.309	-33.757
	$^{ 68 } \text { Ni }$	0.1867	0.0707	0.0096	257.385	-	37.741	9.354	32.472	47.497	-
	$^{ 76 } \text { Zn }$	0.1259	0.0538	0.0098	267.897	-	43.339	11.518	38.199	53.285	-
	$^{ 80 } \text { Ge }$	0.0933	0.0453	0.0102	286.621	-	39.461	5.534	37.382	52.011	-
	$^{ 84 } \text { Se }$	0.036	0	0	305.182	-	35.177	-0.786	36.714	50.480	-
	$^{ 86 } \text { Kr }$	0.0391	0.0115	-0.0001	321.616	-	31.757	-5.509	35.844	49.218	-
	$^{ 90 } \text { Sr }$	-0.0359	0.0014	0.0004	335.402	-	30.242	-8.843	36.896	49.433	-
	$^{ 92 } \text { Sr }$	-0.0602	0.001	0.0001	332.923	-	32.577	-7.047	38.797	51.178	-
	$^{ 94 } \text { Sr }$	-0.0785	0.0064	0.0015	328.873	-	36.262	-3.850	41.302	53.772	-
	$^{ 96 } \text { Zr }$	-0.0497	0.007	0.001	343.525	-	32.847	-8.422	40.649	52.423	-
	$^{ 102 } \text { Mo }$	-0.0547	-0.0037	0.0002	348.313	-	37.368	-5.844	45.250	56.461	-
$_{ 180 }^{ 303 }{ 123 }$	$^{ 4 } \text { He }$	-0.0687	-0.0047	0.0039	14.505	-6.599	-4.950	-7.555	-7.098	-6.595	-
	$^{ 14 } \text { C }$	0.0746	-0.0077	-0.0024	46.759	17.572	19.538	16.988	13.855	16.911	-24.171
	$^{ 16 } \text { O }$	-0.1016	0.0162	0.0008	66.544	19.710	21.112	17.958	14.287	18.070	-26.309
	$^{ 18 } \text { O }$	0.0707	0.003	-0.0052	65.080	23.510	25.464	21.733	18.199	22.449	-30.109
	$^{ 20 } \text { O }$	-0.0719	0.0061	0.0006	61.715	30.311	32.881	28.638	24.859	29.608	-36.910
	$^{ 20 } \text { Ne }$	0.0716	0.0003	-0.0032	81.659	29.612	30.705	26.220	21.785	26.959	-36.211
	$^{ 22 } \text { Ne }$	0.1319	-0.0286	-0.0029	85.328	25.968	28.256	22.963	19.849	25.505	-32.567
	$^{ 24 } \text { Ne }$	0.1627	-0.0513	0.0016	85.207	27.037	30.384	24.391	21.826	27.811	-33.636
	$^{ 26 } \text { Ne }$	0.1789	-0.0575	0.0017	80.844	33.852	38.395	31.839	28.780	35.153	-40.451
	$^{ 28 } \text { Mg }$	0.1911	-0.0589	-0.0001	108.747	25.278	28.599	20.660	19.963	27.307	-31.877
	$^{ 30 } \text { Mg }$	0.2001	-0.0552	-0.0039	104.539	30.567	35.235	26.657	25.495	33.190	-37.166
	$^{ 32 } \text { Si }$	0.2128	-0.0574	-0.0062	131.526	24.086	27.665	17.646	19.170	27.884	-30.685
	$^{ 34 } \text { Si }$	0.2101	-0.0474	-0.0054	129.037	27.127	31.663	20.911	22.507	31.472	-33.726
	$^{ 68 } \text { Ni }$	0.2002	0.0699	0.0052	257.063	-	38.116	9.130	32.343	47.800	-
	$^{ 76 } \text { Zn }$	0.1624	0.0709	0.0138	268.105	-	43.253	10.762	37.784	53.291	-
	$^{ 80 } \text { Ge }$	0.1253	0.0589	0.0108	285.444	-	40.618	6.011	37.612	52.839	-
	$^{ 84 } \text { Se }$	0.087	0.0438	0.0095	302.594	-	37.507	0.854	37.459	52.067	-
	$^{ 86 } \text { Kr }$	0.0689	0.0345	0.0061	317.240	-	35.498	-2.452	37.144	51.694	-
	$^{ 90 } \text { Sr }$	-0.0399	0.0083	0.0009	331.167	-	33.802	-6.008	38.020	51.778	-
	$^{ 92 } \text { Sr }$	-0.0286	0.0035	0.0007	333.026	-	32.608	-7.833	38.486	51.292	-
	$^{ 94 } \text { Sr }$	-0.0493	-0.0014	-0.0005	330.336	-	35.161	-5.806	40.491	53.172	-
	$^{ 96 } \text { Zr }$	-0.03	0	-0.0007	344.060	-	32.534	-9.597	40.203	52.330	-
	$^{ 102 } \text { Mo }$	-0.0685	-0.0048	0.0014	348.990	-	36.953	-7.173	44.724	56.320	-
$_{ 183 }^{ 306 }{ 123 }$	$^{ 4 } \text { He }$	-0.0331	-0.0136	-0.0016	14.182	-5.830	-4.397	-7.268	-7.066	-6.094	-
	$^{ 14 } \text { C }$	0.0733	-0.0223	0.0029	46.932	17.219	19.280	16.588	13.490	16.672	-23.049
	$^{ 16 } \text { O }$	0.0544	-0.0079	0.0001	65.683	20.953	22.231	18.937	15.107	19.039	-26.783
	$^{ 18 } \text { O }$	0.0654	-0.0076	-0.0012	65.021	23.542	25.560	21.656	18.120	22.537	-29.372
	$^{ 20 } \text { O }$	0.0676	0.0006	-0.0038	62.529	28.825	31.622	27.167	23.564	28.482	-34.655
	$^{ 20 } \text { Ne }$	0.0668	-0.0126	0.0002	80.583	30.936	32.112	27.454	22.811	28.129	-36.766
	$^{ 22 } \text { Ne }$	0.0712	-0.0042	-0.0033	84.940	26.885	28.761	23.266	20.082	25.934	-32.715
	$^{ 24 } \text { Ne }$	0.0668	0.0032	-0.0021	85.201	27.815	30.413	24.197	21.654	27.841	-33.645
	$^{ 26 } \text { Ne }$	0.1421	-0.0348	-0.0025	81.061	33.793	38.105	31.310	28.320	34.910	-39.623
	$^{ 28 } \text { Mg }$	0.1691	-0.0517	0.0007	107.515	26.709	30.044	21.877	20.877	28.491	-32.539
	$^{ 30 } \text { Mg }$	0.18	-0.0539	-0.0005	104.414	30.821	35.421	26.576	25.416	33.351	-36.651
	$^{ 32 } \text { Si }$	0.2025	-0.0642	0.0005	129.916	25.658	29.392	19.112	20.209	29.247	-31.488
	$^{ 34 } \text { Si }$	0.2099	-0.06	-0.0041	129.230	26.767	31.482	20.416	22.136	31.340	-32.597
	$^{ 68 } \text { Ni }$	0.2123	0.063	-0.0016	256.790	-	38.446	8.864	32.192	48.071	-
	$^{ 76 } \text { Zn }$	0.1828	0.0766	0.0126	268.903	-	42.618	9.450	37.081	52.930	-
	$^{ 80 } \text { Ge }$	0.1581	0.0731	0.0151	285.077	-	41.054	5.756	37.478	53.194	-
	$^{ 84 } \text { Se }$	0.1175	0.0568	0.0112	300.994	-	39.007	1.650	37.824	53.115	-
	$^{ 86 } \text { Kr }$	0.1011	0.05	0.0108	313.686	-	38.623	-0.019	38.220	53.775	-
	$^{ 90 } \text { Sr }$	0.0643	0.0334	0.009	325.993	-	38.232	-2.277	39.585	54.675	-
	$^{ 92 } \text { Sr }$	0.0457	0.0239	0.006	328.377	-	36.588	-4.574	39.802	53.909	-
	$^{ 94 } \text { Sr }$	-0.033	0.0071	0.0002	329.592	-	35.908	-5.866	40.465	53.742	-
	$^{ 96 } \text { Zr }$	-0.0188	0.0067	-0.001	342.202	-	34.175	-8.767	40.518	53.463	-
	$^{ 102 } \text { Mo }$	-0.0439	-0.0069	0	349.670	-	36.531	-8.502	44.212	56.172	-
$_{ 184 }^{ 307 }{ 123 }$	$^{ 4 } \text { He }$	-0.0152	-0.0037	-0.0005	14.201	-6.005	-4.429	-7.319	-7.113	-6.123	-
	$^{ 14 } \text { C }$	-0.0856	-0.0058	0.0004	47.140	16.865	18.963	16.220	13.161	16.376	-22.870
	$^{ 16 } \text { O }$	-0.0729	-0.0006	-0.0008	65.492	21.101	22.483	19.141	15.277	19.258	-27.106
	$^{ 18 } \text { O }$	0.062	-0.0113	0	65.099	23.423	25.456	21.492	17.975	22.447	-29.428
	$^{ 20 } \text { O }$	-0.1016	0.0162	0.0008	62.871	28.072	31.099	26.571	23.044	28.014	-34.077
	$^{ 20 } \text { Ne }$	0.0631	-0.0148	0.0012	80.302	31.289	32.485	27.768	23.072	28.440	-37.294
	$^{ 22 } \text { Ne }$	0.0688	-0.008	-0.0017	84.918	26.900	28.795	23.230	20.048	25.964	-32.905
	$^{ 24 } \text { Ne }$	0.0716	0.0003	-0.0032	85.546	27.291	29.971	23.671	21.223	27.467	-33.296
	$^{ 26 } \text { Ne }$	0.1319	-0.0286	-0.0029	81.564	33.139	37.383	30.496	27.637	34.292	-39.144
	$^{ 28 } \text { Mg }$	0.134	-0.0304	-0.0029	107.364	27.184	30.228	21.978	20.950	28.643	-33.189
	$^{ 30 } \text { Mg }$	0.1723	-0.0512	-0.0005	104.394	30.883	35.456	26.522	25.369	33.383	-36.888
	$^{ 32 } \text { Si }$	0.1991	-0.065	0.0018	129.322	26.240	30.036	19.671	20.609	29.756	-32.245
	$^{ 34 } \text { Si }$	0.2053	-0.0622	-0.0012	129.084	26.930	31.654	20.489	22.184	31.479	-32.935
	$^{ 68 } \text { Ni }$	0.2164	0.0608	-0.0033	256.676	-	38.577	8.798	32.154	48.175	-
	$^{ 76 } \text { Zn }$	0.1847	0.0762	0.011	269.320	-	42.267	8.871	36.775	52.717	-
	$^{ 80 } \text { Ge }$	0.1602	0.0752	0.0155	285.164	-	41.011	5.480	37.339	53.189	-
	$^{ 84 } \text { Se }$	0.1255	0.0605	0.0115	300.714	-	39.289	1.694	37.843	53.322	-
	$^{ 86 } \text { Kr }$	0.1103	0.055	0.0114	312.662	-	39.539	0.665	38.530	54.388	-
	$^{ 90 } \text { Sr }$	0.076	0.0402	0.0107	324.560	-	39.487	-1.259	40.028	55.500	-
	$^{ 92 } \text { Sr }$	0.0511	0.0258	0.0056	327.140	-	37.671	-3.736	40.153	54.627	-
	$^{ 94 } \text { Sr }$	-0.0399	0.0083	0.0009	328.359	-	36.989	-5.034	40.805	54.461	-
	$^{ 96 } \text { Zr }$	-0.0266	0.0079	-0.001	340.220	-	35.852	-7.332	41.074	54.551	-
	$^{ 102 } \text { Mo }$	-0.0294	-0.0073	-0.0008	350.082	-	36.235	-9.101	43.986	56.025	-

## Summary and conclusion

We have applied the density-dependent cluster model to compute $\alpha$-decay half-lives of the recently synthesized superheavy nuclei with $Z=104$–118. A realistic nucleon–nucleon interaction was folded to generate the microscopic $\alpha$–nucleus potential, which, within the Wentzel–Kramers–Brillouin approximation and Bohr–Sommerfeld quantization, yields the decay widths. Both spherical and deformed daughter configurations were treated, and our results for five isotopes of the superheavy element $Z=123$ show excellent agreement with a variety of established theoretical frameworks. We have further explored the competition between $\alpha$-decay and spontaneous fission, proposing decay chains for the yet-unobserved nuclei ${}^{302\text {--}307}123$. In addition, we examined cluster radioactivity channels of ${}^{300,303,306,307}123$ using five prescriptions: the double-folding model (DFM), the Unified Decay Formula (UDF), the Universal curve (UNIV), Horoi’s law, and the Universal Decay Law (UDL). While DFM, UDF, UNIV, and Horoi consistently favor light-cluster emission and predict $\alpha$ decay as the dominant mode, the UDL uniquely identifies several heavy fragments—for example, ${}^{90}\textrm{Sr}$, ${}^{96}\textrm{Zr}$, and ${}^{102}\textrm{Mo}$—as viable decay channels that can surpass $\alpha$ emission.

## Data Availability

All data generated or analysed during this study are included in this published article.
